# Contribution of oncoproteomics to cancer biomarker discovery

**DOI:** 10.1186/1476-4598-6-25

**Published:** 2007-04-02

**Authors:** William CS Cho

**Affiliations:** 1 Department of Clinical Oncology, Queen Elizabeth Hospital, Room 1305, 13/F, 30 Gascoigne Road, Kowloon, Hong Kong SAR, PR China

## Abstract

Oncoproteomics is the study of proteins and their interactions in a cancer cell by proteomic technologies. Proteomic research first came to the fore with the introduction of two-dimensional gel electrophoresis. At the turn of the century, proteomics has been increasingly applied to cancer research with the wide-spread introduction of mass spectrometry and proteinchip. There is an intense interest in applying proteomics to foster an improved understanding of cancer pathogenesis, develop new tumor biomarkers for diagnosis, and early detection using proteomic portrait of samples. Oncoproteomics has the potential to revolutionize clinical practice, including cancer diagnosis and screening based on proteomic platforms as a complement to histopathology, individualized selection of therapeutic combinations that target the entire cancer-specific protein network, real-time assessment of therapeutic efficacy and toxicity, and rational modulation of therapy based on changes in the cancer protein network associated with prognosis and drug resistance. Besides, oncoproteomics is also applied to the discovery of new therapeutic targets and to the study of drug effects. In pace with the successful completion of the Human Genome Project, the wave of proteomics has raised the curtain on the postgenome era. The study of oncoproteomics provides mankind with a better understanding of neoplasia. In this article, the discovery of cancer biomarkers in recent years is reviewed. The challenges ahead and perspectives of oncoproteomics for biomarkers development are also addressed. With a wealth of information that can be applied to a broad spectrum of biomarker research projects, this review serves as a reference for biomarker researchers, scientists working in proteomics and bioinformatics, oncologists, pharmaceutical scientists, biochemists, biologists, and chemists.

## Background

More than 11 million people are diagnosed with cancer every year. It is estimated that there will be 16 million new cases every year by 2020. From a total of 58 million deaths worldwide in 2005, cancer accounts for 7.6 million (or 13%) of the global mortality. Deaths from cancer in the world are projected to continue rising, with an estimated 9 million people dying from cancer in 2015 and 11.4 million dying in 2030.

As an important biological indicator of cancer status and progression for the physiological state of the cell at a specific time, biomarkers represent powerful tools for monitoring the course of cancer and gauging the efficacy and safety of novel therapeutic agents. They can have tremendous therapeutic impact in clinical oncology, especially if the biomarker is detected before clinical symptoms or enable real-time monitoring of drug response. There is a critical need for expedited development of biomarkers and their use to improve diagnosis and treatment for cancer. Malignant transformation involves alterations in protein expression with subsequent clonal proliferation of the altered cells. These alterations can be monitored at the protein level, both qualitatively and quantitatively. Protein signatures in cancer provide valuable information that may be an aid to more effective diagnosis, prognosis, and response to therapy.

The recent progress of proteomics has opened new avenues for cancer-related biomarker discovery. Advances in proteomics are contributing to the understanding of pathophysiology of neoplasia, cancer diagnosis, and anticancer drug discovery. With the advent of new and improved proteomic technologies such as the development of quantitative proteomic methods, high-resolution, high-speed, high-throughput, high-sensitivity mass spectrometry (MS) and proteinchip, as well as advanced bioinformatics for data handling and interpretation, it is possible to discover biomarkers that are able to reliably and accurately predict outcomes during cancer treatment and management. Besides, the newer technologies provide higher analytical capabilities, employing automated liquid handling systems, fractionation techniques and bioinformatics tools for greater sensitivity and resolving power, more robust and higher throughput sample processing, and greater confidence in analytical results can be obtained. Oncoproteomics offers cutting-edge capabilities to accelerate the translation of basic discoveries into daily clinical practice. Continued refinement of techniques and methods to determine the abundance and status of proteins holds great promise for the future study of cancer and the development of cancer therapies [1,2].

## Current tumor markers

Early diagnosis of cancer is difficult because of the lack of specific symptoms in early disease and the limited understanding of etiology and oncogenesis. For example, blood tumor markers for breast cancer such as cancer antigen (CA) 15-3 are useless for early detection because of low sensitivity. Therefore measurement of carcinoembryonic antigen (CEA) and HER-2 in abnormal nipple discharge has been approved for diagnosis of breast cancer in some countries [3]. More than 98% of cervical cancer is related to human papillomavirus (HPV) infection. The identification and functional verification of host proteins associated with HPV E6 and E7 oncoproteins may provide useful information for the understanding of cervical carcinogenesis and the development of cervical cancer-specific markers [4]. For hepatocellular carcinoma (HCC), the common method of screening high risk patients by alpha-fetoprotein (AFP) and ultrasonography has been shown to result in earlier detection and consequently more easily treatable tumors and longer survival. Of the other tumor markers, the newer high sensitive des-gamma-carboxy-prothrombin has been found to be useful. In addition, the AFP fractions L3, P4/5, and the +II band are highly specific for HCC. Among routinely assayed tumor markers in the laboratory, CA-125 is more sensitive for HCC than AFP but far less specific [5]. Currently available screening tests for ovarian cancer include CA-125, transvaginal ultrasound, or a combination of both. CA-125 has provided a useful serum tumor marker for monitoring response to chemotherapy. A rapid fall in CA-125 during chemotherapy predicts a favorable prognosis and can be used to redistribute patients on multiarmed randomized clinical trials. Prostate-specific antigen (PSA) is the most important tumor marker in all solid tumors, indispensable in the management of prostate cancer [6]. However, most currently available screening tests for cancers lack high sensitivity and specificity to be useful in screening the general population, so the differentiation between some benign and malignant tumors is still a clinical challenge. The advent of oncoproteomics has provided the hope of discovering novel biomarkers for use in the screening, early diagnosis, and prediction of response to therapy (Table 1).

**Table 1:** Comparison of proteomic biomarkers and current tumor markers

**Cancer**	**Proteomic biomarkers**			**Current tumor markers**		
		
	**Sensitivity**	**Specificity**	**Reference**	**Markers**	**Sensitivity**	**Specificity**
Bladder	80%	90–97%	[87]	NMP22	31%	95%
Breast	93%	91%	[88]	CA 15-3	63%	80–88%
Colorectal	91%	93%	[89]	CEA	43%	****
Gastric	83%	95%	[90]	CEA	49%	****
Liver	94%	86%	[91]	AFP	50%	90%
Lung	87%	80%	[92]	Cyfra21-1	63%	94%
Ovarian	83%	94%	[93]	CA-125	57%	****
Pancreatic	78%	97%	[94]	CA 19-9	72%	****
Prostate	83%	97%	[95]	PSA	86%	20–34%

## Diagnostic biomarkers

Diagnostic oncoproteomics is the application of proteomic techniques for the diagnosis of malignancies. The early detection of cancer has a potential to dramatically reduce mortality. The thermostable fractions of serum samples from patients with ovarian, uterus, and breast cancers, as well as samples from benign ovarian tumor were analyzed using two-dimensional gel electrophoresis (2-DE) combined with matrix-assisted laser desorption/ionization time-of-flight (MALDI-TOF)/TOF MS. Of them, alpha-1-acid glycoprotein and clusterin were expressly down-regulated in breast cancer, whereas transthyretin was decreased specifically in ovarian cancer. Apolipoprotein A-I forms have decreased spot volumes, while haptoglobin alpha1, in contrast, was elevated in several tumors. Serum thermostable fraction may be recommended as a good tool for medium and small protein investigation, in particular, by 2-DE [7].

### Bladder cancer

Celis and co-workers have utilized 2-DE and MS analysis to identify differential protein expression between bladder cancer and healthy tissue including squamous cell carcinomas versus normal urothelium, which has defined some of the steps involved in the squamous differentiation of the bladder transitional epithelium [8]. On the other hand, making use of 2-DE and MS/MS, Sheng *et al *recently discovered that fatty acid binding proteins, annexin V, heat shock protein (Hsp) 27, and lactate dehydrogenase were associated with bladder cancer. They also found altered expression of a group of proteins in bladder cancer that have not been documented previously, including annexin I, 15-hydroxyprostaglandin dehydrogenase, galectin-1, lysophospholipase, and mitochondrial short-chain enoyl-coenzyme A hydratase 1 precursor [9].

### Breast cancer

Isotope-coded affinity tag tandem MS allows for qualitative and quantitative analysis of paired protein samples. Alpha2-HS-glycoprotein was under-expressed in nipple aspirate fluid from tumor-bearing breasts, while lipophilin B, beta-globin, hemopexin, and vitamin D-binding protein precursor were over-expressed [10].

### Colorectal cancer

Six proteins (ANXA3, BMP4, LCN2, SPARC, MMP7, and MMP11) were found to be over-expressed in colorectal tumoral tissues by using immunoblotting and tissue microarray analysis. Two of them (LCN2 and MMP11) were clearly over-expressed in late Dukes stages (B and C) [11]. To identify proteins with colorectal cancer (CRC)-specific regulation, comparative 2-DE of individual-matched normal and neoplastic colorectal tissue specimens was performed. Endocrine cell-expressed protein secretagogin exhibited a marked down-regulation in CRC tissues. This finding may represent the basis for the clinical application of secretagogin as a biomarker for a distinct subgroup of CRCs [12].

### Esophageal cancer

Using agarose 2-DE and agarose 2-D difference gel electrophoresis (DIGE), a number of proteins with altered expression between primary esophageal cancer and adjacent non-cancer tissues have been identified. Among them, periplakin was significantly down-regulated in esophageal cancer, which was confirmed by immunoblotting and immunohistochemistry. These results suggested that periplakin could be a useful marker for the detection of early esophageal cancer and the evaluation of tumor progression [13,14].

### Gastrointestinal stromal tumor

To investigate the molecular characteristics of gastrointestinal stromal tumor (GIST) according to mutation type, protein expression profiles in GIST were analyzed using 2-DE and MALDI-TOF MS. Among the 15 proteins differently expressed according to the mutation status, over-expression of 5 proteins (annexin V, high mobility group protein 1, C13orf2, glutamate dehydrogenase 1, and fibrinogen beta chain) and decreased expression of RoXaN correlated with a higher tumor grade. These findings suggested that differential protein expression could be used as diagnostic biomarkers [15].

### Glioma

Application of direct tissue MALDI-TOF MS to human brain tumors identified protein patterns that distinguished primary gliomas from normal brain tissue and one grade of gliomas from another, with high sensitivity and specificity [16].

### Hepatocellular carcinoma

Comparative proteomic analysis was used to search for characteristic alterations in the sera of HCC patients who had undergone curative radiofrequency ablation treatment subjected to 2-DE, and the proteins were identified by MS based on MALDI-TOF/TOF analysis and public database searches. The statistical analysis suggested that 4 proteins decreased after treatment, including pro-apolipoprotein, alpha2-HS glycoprotein, apolipoprotein A-IV precursor, and PRO1708/PRO2044 (the carboxy terminal fragment of albumin). Seven proteins were increased after treatment, including leucine-rich alpha2-glycoprotein and alpha1-antitrypsin. These data provided candidate biomarkers for the development of diagnostic and therapeutic tools [17]. On the other hand, 2-D DIGE combined with nano flow liquid chromatography (LC) tandem MS was employed to investigate differentially expressed proteins in HCC. 14-3-3γ protein was found to be up-regulated in HCC. 14-3-3 isoforms has been linked to carcinogenesis because they are involved in various cellular processes such as cell cycle regulation, apoptosis, proliferation, and differentiation [18]. Surface-enhanced laser desorption/ionization (SELDI)-TOF MS was also performed to identify differentially expressed proteins in HCC serum using weak cation exchange proteinchips. Protein characterization was performed by 2-DE separation and nano flow LC-MS/MS. Complement C3a was detected as differentially expressed in patients with chronic hepatitis C and hepatitis C virus-related HCC. This result was further validated by PS20 chip immunoassay and Western blotting [19]. Besides, the use of proteinchip technology in combination with tissue microdissection has identified ferritin light subunit, adenylate kinase 3 alpha-like 1, and biliverdin reductase B in HCC [20].

### Leukemia

DotScan microarray (a cluster of differentiation antibody microarray) has been developed to enable an extensive immunophenotype obtained for a suspension of leukocytes in a single analysis. The antibody microarray is printed as microscopic (10 nL) dots on a nitrocellulose film on microscope slide. Cells are captured by the immobilized antibodies and a dot pattern is recorded with an optical array reader giving the immunophenotype of leukemia. Procedures are developed to enable diagnosis of myeloid leukemia by comparison of the dot pattern obtained from an unknown blood sample with a library of consensus patterns for common leukemia [21].

### Lung cancer

Based on the assumption that proteins can emanate from tumor to serum, Maciel *et al *investigated whether serum proteins could discriminate lung adenocarcinoma patients from healthy donors. Results of 2-DE/MALDI-TOF showed 5 up-regulated proteins (immunoglobulin lambda chain, transthyretin monomer, haptoglobin-alfa 2, and 2 isoforms of serum amyloid protein) and 1 down-regulated protein (fragment of apolipoprotein A-I) in lung adenocarcinoma patients [22].

### Lymphoma

Fan *et al *making use of two-way hierarchical clustering analysis of the protein expression profiles differentiated reactive follicular hyperplasia, follicular lymphoma, and Burkitt lymphoma, with 5 major clusters of differentially expressed protein peaks for molecular classification of B cell lymphoma subtypes. They identified histone H4 as a potential differentially expressed protein marker that seemed to distinguish grade 1 from grade 3 follicular lymphoma [23].

### Nasopharyngeal carcinoma

Unfractionated whole sera of newly diagnosed Malaysian Chinese patients with advanced nasopharyngeal carcinoma were subjected to 2-DE and image analysis, ceruloplasmin showed higher expression. The enhanced expression of ceruloplasmin in the patients' sera was confirmed by competitive enzyme-linked immunosorbent assay (ELISA) [24].

### Ovarian cancer

Identified by MALDI-TOF MS and validated by Western blotting, haptoglobin precursor significantly up-regulated while transferrin precursor significantly down-regulated in grade 3 ovarian cancer patients. Changes in serum expression of haptoglobin correlated with the change of CA-125 levels before and after chemotherapy [25]. Of great significance, the technique worked well on patients with early stage disease, offering the prospect of earlier diagnosis which would greatly enhance the chance of successful treatment outcome. This has led to the development of a commercial test, termed OvaCheck, for diagnosis of ovarian cancer.

### Pancreatic cancer

The survival rate of pancreatic cancer patients is the lowest among those with common solid tumors, and early detection is one of the most feasible means of improving outcomes. Proteomic analysis combining 2-DE and MS successfully identified 154 potential serum markers for pancreatic cancer. Of these, fibrinogen γ, a protein associated with the hypercoagulable state of pancreatic cancer, discriminated cancer from normal sera. Fibrinogen γ was subsequently confirmed to be over-expressed in pancreatic cancer sera by enzymatic analysis and tissue by immunohistochemistry relative to normal pancreas, thus it is a potential tumor marker in pancreatic cancer [26]. Besides, a PowerBlot analysis with more than 900 well-characterized antibodies was performed with tissue specimens from patients with chronic pancreatitis, pancreatic adenocarcinoma, and normal pancreas. A large number of proteins are differentially expressed in the chronic pancreatitis and pancreatic adenocarcinoma compared with the normal pancreas. Among them, expression analysis of UHRF1, ATP7A, and aldehyde oxidase 1 in combination could potentially provide a useful additional diagnostic tool for fine-needle aspirated or cytological specimens obtained during endoscopic investigations [27].

### Prostate cancer

A promising prostate cancer biomarker identified by 2-DE and MS is annexin I. Studies have already confirmed that annexin I is under-expressed in a majority of early stage prostate cancer. Other non-gel-based proteomic technologies that may have improved sensitivity as compared to 2-DE have recently been developed, one of the examples is the ProteomeLab PF 2-D (Beckman Coulter Inc, Fullerton, CA, USA). The goal of most proteomic studies is to identify biomarkers that can be measured by ELISA or immunohistochemistry. Improvements in proteomic technology are changing this paradigm because there are now efforts to develop proteomic technologies directly into clinical diagnostic tests, an example of these technologies is SELDI-TOF MS. Using this technology combined with pattern recognition based bioinformatics tool, discriminatory spectrum proteomic profiles were generated which could help discriminating men with prostate cancer from those with benign prostate [28].

### Renal cancer

It is possible to define specific protein patterns in the serum of renal cancer. Several proteins have been identified by SELDI, including serum amyloid alpha [29].

### Urothelial carcinoma

Using capillary electrophoresis-coupled MS to obtain polypeptide patterns from urine samples of patients with urothelial carcinoma and healthy volunteers, a prominent polypeptide from the diagnostic pattern for urothelial carcinoma was identified as fibrinopeptide A (a known biomarker of ovarian cancer and gastric cancer). Validation of a highly specific biomarker pattern for urothelial carcinoma in a large group of patients with various urological disorders can be used in the diagnosis of other diseases that are identified in urine samples or in other body fluids [30].

## Screening biomarkers for cancers

Population proteomics is an applied proteomics subdiscipline engaging in the investigation of human proteins across and within populations to define and better understand protein diversity. Population proteomics focuses on interrogation of specific proteins from a large number of individuals, utilizing top-down, targeted affinity MS approaches to probe protein modifications. Deglycosylation, sequence truncations, side-chain residue modifications and other modifications have been reported for myriad of proteins, yet little is known about their incidence rate in the general population. Such information can be gathered via population proteomic studies, and would greatly aid the biomarker discovery efforts. Identification of novel protein modifications is also expected from such large-scale population proteomic studies, expanding the protein knowledge database. In regards to tumor biomarkers, their validation via population proteomic approaches is advantageous as MS detection is used both in the discovery and validation process, which is essential for the detection of structurally modified tumor biomarkers [31].

For instance, screening of the head and neck carcinoma patients with the proteomics-based autoantibody-mediated identification of antigens technology yielded a set of tumor-associated antigens. Expression of cytokeratin (CK) 8 correlated positively with malignancies of the head and neck areas. CK8 is an attractive marker molecule for a differentiated diagnosis between leukoplakia with head and neck carcinomas, which possesses notedly improved specificity as compared with panCK and CK13 [32].

## Biomarkers in cancer development

The action of regulatory circuits, cross-talk between pathways and the non-linear reaction kinetics of biochemical processes complicate the understanding and prediction of the outcome of intracellular signaling [33]. Like normal cells, most cancer cells use multiple redundant intracellular signaling pathways to ensure the maintenance and viability of functions critical to their survival. Thus, cellular pathways that are integral to cell function, survival, proliferation, and receptor expression are potential targets for therapeutic intervention, with epidermal growth factor receptor (EGFR) signaling pathway as one of the good examples. Clinicians might recommend combinations of molecularly targeted agents and other therapies on the basis of an individual patient's proteomic profile [34].

Understanding the molecular basis of the biochemical pathways involved in carcinogenesis can facilitate the integration of diagnosis, anticancer drug discovery, and therapy for cancer. The powerful -omic technologies have enabled the identification of key biomarkers and signaling molecules associated with cell growth, cell death, and cellular metabolism. A realistic model of cellular regulation based on current knowledge indicates that many interacting networks operate at the epigenetic, transcriptional, translational, and post-translational levels, with feedback between the various levels. Protein-protein and protein-DNA interactions help to define which genes may be activated in a particular cell, and determine whether external cues cause activation or repression. Proteomic technologies will ultimately characterize information-flow through the protein circuitry that interconnects the extracellular microenvironment to the serum or plasma macroenvironment through the intracellular signaling systems and their control of gene transcription. The purpose of differential and functional proteomics is to obtain this information which will lead to improved understanding of cellular pathways and their inter-relationships in cells and living organisms. The nature of this information can be a cause or a consequence of disease processes and how patients respond to therapy [35]. New technologies, such as proteomics using MS, high-density DNA or oligonucleotide microarrays, and chromatin immunoprecipitation provide new and exciting tools for deciphering the pathways and proteins controlling gene expression. Analysis of these pathways offers new insight that aids targeted drug development which promises to revolutionize clinical practice [36].

For example, changes in protein expression levels revealed a significantly enhanced glycolytic pathway (Warburg effect), a decreased gluconeogenesis, a suppressed glucuronic acid pathway, and an impaired tricarboxylic acid cycle in CRC using MALDI-TOF/TOF MS. Observed changes in protein abundance were verified by 2-D DIGE. These changes reveal an underlying mechanism of colorectal tumorigenesis in which the roles of impaired tricarboxylic acid cycle and Warburg effect may be critical [37]. On the other hand, the study of the human colon cancer proteome represents a step toward to a better understanding of the metabolomics of colon cancer at early stages confined to the intestinal wall. A shift from beta-oxidation, as the main source of energy, to anaerobic glycolysis was observed owed to the alteration of nuclear- versus mitochondrial-encoded proteins and other proteins related to fatty acid and carbohydrate metabolism. Lower capacity for Na(+) and K(+) cycling was found, and operativity of the apoptosis pathway (especially the mitochondrial one) was concluded [38]. It is suggested that CRC may be prevented by changes in diet, and vegetable consumption has been demonstrated to have a protective effect. Until now, little is known about the effects of vegetable consumption at the proteome level. Six proteins were identified by MALDI-TOF MS, including myosin regulatory light chain 2, carbonic anhydrase I, high-mobility group protein 1, pancreatitis-associated protein 3, glyceraldehyde-3-phosphate dehydrogenase, and ATP synthase oligomycin sensitivity conferral protein. Alterations in the levels of these proteins agreed with a role in the protection against colon cancer. The observed altered protein levels therefore provided support for the protective effects of vegetables against CRC [39]. Another good example is the Epstein-Barr virus (EBV) study, EBV-encoded latent membrane protein 1 (LMP1) can activate NF-κB, activator protein-1, and Janus kinases/signal transducer and activation of transcription factors pathways. Combining the novel strategy of phosphoprotein enrichment with proteomic technology to elucidate the signaling cascade activated by LMP1, it was reported that LMP1 could increase the quantity of total phosphoproteins. The other proteins, including annexin A2, Hsp27, stathmin, annexin I, basic transcription factor 3, and porin, were novel signaling molecules or targets with no previously known function in LMP1 signal transduction. The method used has proven to be suitable for the identification of molecules involved in various signaling pathways [40].

A comprehensive understanding of the metastatic pathways is crucial for the improvement of the limited therapeutic weapons currently at disposal. Some studies have addressed this subject very recently, biochemical confirmation of cleavage of the potential substrates was performed and the cleavage sites were identified by MALDI-TOF. Using proteomics and metabolic profiling, sorbitol (a component of an alternative glycolysis pathway) was significantly elevated at 5.4-fold in renal cell carcinoma patients as compared to the controls. This finding may be used to influence the choice of optimal therapy [41]. Overall and Dean discovered and confirmed that CTGF, galectin-1, death receptor-6, Hsp90alpha, procollagen C-proteinase enhancer protein, chemokine fractalkine, and cystatin C were novel MT1-matrix metalloproteinases (MMP) or MMP-2 substrates. These sophisticated cellular control functions highlight new intervention points in multiple pathways to treat early stage cancer [42]. On the other hand, analysis of 5 independent studies comprised of greater than 1 × 10^6 ^genomic sequences and greater than 1,000 proteins revealed that the cytoskeletal-associated protein alpha-actinin was increased at both the mRNA and protein level in metastatic breast, prostate, and skin cancer cells. Spatial analysis of alpha-actinin expression revealed that it was amplified 8-fold in the leading pseudopodium compared to the cell body compartment of migrating cells. These findings indicated that amplification of alpha-actinin and its localization to the leading pseudopodium were potential biomarkers of cancer progression to a more metastatic phenotype [43].

## Biomarkers for targets in cancer therapy

Results from genomic and proteomic studies are eagerly awaited for selecting patients, avoiding the use in non-targeted situation and reducing the cost of treatments. One of the major contributions proteomics has made to the medical and pharmaceutical communities is the identification of potential drug targets. Many cancers are characterized by alternations in certain signaling pathways and identification of the aberrant pathway in a particular patient allows for targeted therapy to that specific pathway. For example, epithelial ovarian cancer is often characterized by activation of EGFR signaling pathway, and targeted therapies including monoclonal antibodies, such as cetuximab and small molecule inhibitors such as gefitinib are either in clinical use or under clinical trial for different stages of cancer. Similarly, the c-Kit and platelet-derived growth factor receptor inhibitor, Imatinib, has shown remarkable success in chronic myeloid leukemia and GIST, cancers that are maintained by the activation of these receptor tyrosine kinases.

Proteinchip has been employed to measure enzyme activity of secreted and membrane proteomes of cancer cell lines, and are now being used to measure kinase activity via specific detection of phosphoproteins [44]. It is believed that phosphorylation of various proteins, such as cyclin E, cyclin D, p27, IκB-α, and STAT1, allows them to be ubiquitinated and marked for proteolysis by the proteasome complex. On the other hand, phosphorylation of other proteins, such as c-Fos and c-Jun, prevents their ubiquitination. This further indicates a direct involvement of the proteasome in cell proliferation and cell cycling processes. The first selective proteasome inhibitor, bortezomib (Velcade), has been synthesized for recognizing the potential of a proteasome inhibitor as a novel cancer therapeutic, and found out the relationship between the proteasome, NF-κB and multiple myeloma. Proteasomes are large multi-subunit protease complexes that are localized in the nucleus and cytosol which selectively degrade intracellular proteins. They play a major role in the degradation of many proteins involved in cell cycling, proliferation, and apoptosis. The ubiquitin-proteasome pathway involves in the breakdown of short-lived abnormal proteins which result from oxidative stress and mutations that might otherwise disrupt normal cellular homeostasis. The reactive oxygen species promote partial unfolding of the proteins, exposing its hydrophobic domains to proteolytic enzymes of 20S complex. Ubiquitin-medicated pathway in cancer includes ubiquitin-medicated down-regulation of receptor tyrosine kinases in cancer, control of the cell cycle by the ubiquitin system, regulation of DNA repair by the ubiquitin system and its implication in cancer. It has been shown that actively proliferating cancer cells are more susceptible to the action of proteasome inhibitors than non-cancerous cells. Constitutively active NF-κB pathway is common in several solid tumors and proteasome inhibitors block this activation and make cancer cells more susceptible to radiation therapy and chemotherapeutic agents.

### Breast cancer

The monoclonal antibody inhibitor of HER-2, trastuzumab (Herceptin), has been used successfully as monotherapy and in combination with chemotherapy in women with HER-2 over-expressing metastatic breast cancer [45-48]. Besides, hormone receptors have been used as reliable predictive factors for response to endocrine therapy. Other biomarkers have been investigated to select patients with tumors hormone receptors-positive but unresponsive to endocrine therapy [3]. Proteomics-based studies have also widened our knowledge of transforming growth factor-β-dependent regulation of cell proliferation, apoptosis, DNA damage repair and transcription. This leads to better understanding of the transforming growth factor-β role in human breast tumorigenesis and opens the avenue for the development of novel anticancer treatments and drugs, with some of the drugs already entering clinics [49].

### Colorectal cancer

Bevacizumab receives European Union approval for the first-line treatment of metastatic CRC in combination with irinotecan- or 5-FU-based chemotherapy. Bevacizumab prevents interaction of VEGF with VEGFR1 (FLT-1) and VEGFR2 (KDR) on the surface of endothelial cells to inhibit angiogenesis. Bevacizumab is used for the first-line and second-line treatment of metastatic CRC. Besides, cetuximab became the first EGFR-targeting monoclonal antibody approved for use in metastatic CRC in 2004. Cetuximab is an IgG1 monoclonal antibody that specially targets the EGFR with high affinity and competitively inhibits endogenous ligand binding. It binds exclusively EGFR and its heterodimers, blocks receptor dimerisation, tyrosine kinase phosphorylation, and signal transduction. Cetuximab has shown good efficacy in combination with irinotecan in CRC that had previously progressed on irinotecan-based therapy. Cetuximab plus irinotecan and various schedule of 5-FU/FA have shown efficacy in a first-line setting [50].

### Hepatocellular carcinoma

By reverse transcriptase-polymerase chain reaction, a 1,741 bp cDNA encoding a protein that is differentially expressed in HCC have been isolated. This novel protein was identified by proteomic analysis and was designated as Hcc-2, which is up-regulated in poor-differentiated HCC but unchanged in well-differentiated HCC. This work demonstrated that an integrated proteomic and genomic approach could be a very powerful means of discovering potential diagnostic and therapeutic protein targets for cancer therapy [51].

### Prostate cancer

The identification of antigens expressed by prostate tissue and/or prostate cancer that are recognized by T cells or antibodies creates opportunities to develop novel immunotherapeutic approaches including tumor vaccines. Proteins expressed in prostate cancer including PSA, prostatic acid phosphatase, and prostate membrane antigen have been used as immunologic targets for immunotherapy [52].

## Biomarkers for therapeutic response monitoring and prognosis

Prognostication and the variability of tumor responses to radio-/chemo-therapeutic agents is a topic of major interest in current cancer research. The advances in proteomic research will lead to a plethora of new molecular markers, which are likely to be correlated with disease activity, progression, and survival. Pharmacoproteomics, a novel discipline that investigates the protein expression in tumor cells and the response to anticancer agents, evaluation of radio-/chemo-therapy particularly for the characterization of drug-resistance mechanisms, will be instrumental in developing optimal anticancer regimens for patients. Mechanisms mediating drug-resistance are multifaceted. Rapid developments in proteomic technologies have made it possible to simultaneously identify multiple proteins involved in drug refractory cancers. Advances in the knowledge of dysregulation of key molecular pathways in cancer cells have enabled techniques to be developed that can profile tumor cells for their genetic background, allowing selection of anticancer agents on an individual basis. The next generation of anticancer treatments might therefore be tailored according to the molecular alterations identified in tumor cells of individual patients [53,54].

### Bladder cancer

At least 50% of patients with a history of bladder cancer have recurrences, so rigorous surveillance is necessary. The noninvasive point-of-care assay for elevated urinary nuclear matrix protein NMP22 can increase the ability to detect recurrent bladder cancer [55].

### Breast cancer

The presence of progesterone receptor (PR) in estrogen receptor (ER) positive breast cancer is associated with a good prognosis, and indicates that tumors are likely to respond to tamoxifen. However, ER+/PR- tumors respond less well. Owing to the fact that current proteomic methods are hampered in the examination of most primary human tumor samples by the extreme tissue heterogeneity, laser capture microdissection was used to isolate tumor cells and developed a sample pooling strategy to analyze small sample protein lysates. The differentially displayed proteins included decreased cytochrome b5 and transgelin, and more abundant CRABP-II, cyclophilin A, neudesin, and hemoglobin in ER+/PR+ tumors versus ER+/PR- providing a possible explanation for differential susceptibility against tamoxifen as a result of deregulated cytochrome b5-dependent metabolism [56]. Besides, SELDI-TOF MS showed that a high level of cytosolic ubiquitin and a low level of ferritin light chain were associated with a good prognosis in breast cancer. Differential expressions of the two proteins were further confirmed by Western blotting analysis and immunohistochemistry [57]. Furthermore, multidrug resistance is a major obstacle to successful breast cancer treatment. Following 2-DE and MALDI-TOF MS analysis, functional validation showed that the elevated 14-3-3σ expression contributed considerably to the observed drug resistance in MCF7/AdVp3000 cells. Its altered expression in tumors might cause clinical resistance to chemotherapy [58].

### Hepatocellular carcinoma

Employing 2-DE and MS/MS, 3 chaperone members (Hsp27, Hsp70, and glucose-regulated protein 78) were found to be over-expressed in HCC tissues. Confirmed by Western blotting and immunohistochemistry, no significant association of Hsp70 with any pathologic features was observed. The HCC proteome analysis revealed that in response to the stressful cancerous microenvironment, tumor cells strived to increase the expression of chaperone proteins for cyto-protective function and to enhance tumor growth and metastasis [59].

### Leukemia

Using the comparative proteomic approach, several Hsps known to complex Bcr-Abl were over-expressed in imatinib resistant chronic myelogenous leukemia cells, showing a possible involvement of these proteins in the mechanism of resistance. HnRNPs also resulted in being up-regulated in imatinib resistant cells. These proteins have been shown to be strongly and directly related to Bcr-Abl activity [60].

### Lymphoma

Pharmacogenetics and pharmacoproteomics have been instrumental in developing optimal chemotherapeutic regimens for patients with non-Hodgkin's lymphoma [61]. Correlating the protein expression profiles by 2-DE with clinical staging of B cell chronic lymphocytic leukemia patients, Hsp27 was found to be over-expressed in patients with shorter survival times. Down-regulation of thioredoxin peroxidase 2 and protein disulfide isomerase also correlated to decreased survival times. Identification of these proteins is of particular prognosis value in B cell chronic lymphocytic leukemia patients [62].

### Nasopharyngeal carcinoma

Using SELDI-TOF MS analysis, Cho *et al *have identified serum biomarkers (two isoforms of serum amyloid A protein) that were useful to monitor relapse of nasopharyngeal carcinoma. Monitoring the patients longitudinally for serum amyloid A level both by proteinchip and immunoassay showed a dramatic increase, which correlated with relapse and a drastic fall correlated with response to salvage chemotherapy [63]. Using similar approach, 13 other serum biomarkers (including ITIH4 and PF4) that are associated with active disease or chemotherapy response in nasopharyngeal carcinoma patients were further discovered [64,65].

### Ovarian cancer

Analyzed by peptide fragment matching and MS/MS, cisplatin caused notably increase expressions of some proteins in ovarian cancer, including tropomyosin family, actin family, triosephosphate isomerase family, and Hsp60, while expressions of some proteins in the enolase family decreased. Those proteins were involved in cellular energy metabolism, transformation, apoptosis, and morphologic maintenance suggested that alterations of the physiological processes might be involved in antitumor mechanism of cisplatin [66]. Besides, more than 30 serum markers have been evaluated alone and in combination with CA-125 by different investigators. Some of the most promising proteins include HE4, mesothelin, M-CSF, osteopontin, kallikrein, and soluble EGF receptor. Serum markers may improve the sensitivity of detecting recurrent disease and facilitate earlier detection of ovarian cancer [67].

## Challenges

Identification of large numbers of proteins from complex biological samples is a continuing challenge in the area of quantitative proteomics. When coupled with 2-D-LC/nano- electrospray ionization-MS, this method allows enhanced protein identification when tested on samples from prokaryotic and eukaryotic sources. The sample complexity can be effectively reduced with corresponding increases in protein identification using the multistep method. This strategy represents a potentially powerful technique for large-scale qualitative and quantitative proteome research [68].

New challenges arise in large scale proteomic profiling when dealing with complex biological mixtures such as mammalian cell lysate. The approach of protein separation prior to the shotgun multidimensional protein identification technology was explored. Using the PF 2-D ProteomeLab system, the mammalian cancer cell lysate was fractionated and the distribution of molecular weight, isoelectric point, and cellular localization of the eluted proteins were analyzed. Sample complexity was reduced by protein fractionation and the possibility of detecting proteins with lower abundance in the complex protein mixture was increased [69]. Another difficult task is to identify protein from formalin-fixed paraffin-embedded specimens. Recent research has successfully overcome this bottleneck, which has significant implications for tandem MS-based proteomics of vast repositories of archival primary tissue samples for disease-related discovery research [70].

The biological variability among patient samples as well as the huge dynamic range of biomarker concentrations is also the current main challenge to deduce diagnostic patterns that are unique to specific cancer states [71]. Specimen manipulations such as sample collection, pipetting, and diluting contribute to pre-analytical variables. In biomarker research, samples are usually collected from multiple sites and randomly divided into discovery (training) sets and validation (testing) sets. Differences in sample collection, handling or storage, and profiling techniques, may influence the protein profile obtained from a given sample [72-74]. Wang *et al *introduced a simple "single-tube" preparation protocol appropriate for small protein samples using the organic cosolvent, trifluoroethanol, to circumvent the loss of sample by facilitating both protein extraction and protein denaturation without requiring a separate cleanup step [75]. Anyway, issues regarding biological variation, pre-analytical variables and analytical variability must be tackled.

Although proteomics has proved its promise for biomarker discovery, further work is still required to enhance the performance and reproducibility of established proteomic tools before they can be routinely used in clinical laboratory. It is becoming increasingly recognized that reproducibility and validation of tumor biomarkers should be addressed carefully, as should their origin and identity. An extremely important aspect of the Human Proteome Organization is to provide standardization of techniques, particularly once proteome analyses become routine use in the clinical setting. A number of technical obstacles remain before routine proteomic analysis can be achieved in the clinic. However, the standardization of methodologies and dissemination of proteomic data into publicly available databases is starting to overcome these hurdles. Furthermore, the cost is also a precluding factor for the widespread use of proteomics in clinical laboratory. Most proteomic technologies use complex instrumentation, critical computing power, and expensive consumables. Another major challenge will be the integration of proteomic with genomic and metabolomic data and their functional interpretation in conjunction with clinical results and epidemiology [76].

On the other hand, life sciences are rapidly changing from disciplines that were dealing with relatively small datasets to research areas bombarding with large and huge data sets. When proteomic technology matures, we may even see datasets with more than a million variables. Due to the large numbers of variables, exhaustive search that would guarantee finding the best subset cannot be implemented. Because of these difficulties, many studies reported in the literature pretty much neglect this step and apply more or less arbitrary selection of features used to build classification models. Usual approach is to find an ordered list of features using simple univariate methods like ANOVA and then use some of the features from the top of the list. Such a univariate approach not only neglects correlations between variables, but also results in removing from important discriminatory information. Traditional univariate approach, which has dominated life sciences for a very long time, is no longer adequate. Different approaches are necessary and multivariate analysis should become a standard one.

Another limiting step in the biomarker pipeline is assay development. If one can discover hundreds of candidates at the tissue or blood level, the problem is going to be assaying the hundreds of candidates in hundreds of samples. Although ELISA is the standard clinical assay for low-abundant proteins, it is too expensive to implement on large scale. Therefore, it is suggested to use the method of stable isotope standards and capture by anti-peptide antibodies and multiple-reaction-monitoring MS. Some researchers are investigating this method to develop multiplexed assays for several potential biomarkers [77].

## Perspectives

Genomics offers the opportunity to examine gene expression or the variation in gene sequence, whereas proteomics encompasses evaluation of protein expression, activation, modification, degradation, and ambitiously targets protein function. The human proteome, due to the enormity of post-translational permutations that result in large numbers of isoforms, is much more complex than the genome and alterations in cancer which can occur in ways that are not predictable by translational analysis alone. Conceptually, proteomics bears the advantage of incorporating both post-translational modifications (PTMs) as well as host factors. This is thought to be important in factors influencing survival such as chemo-resistance. Proteomic approach avoids overlooking PTMs not detected at gene level and the limited correlation between transcript and protein levels. Phosphorylation is a dynamic PTM that regulates the function of many proteins, and is intimately involved in cellular signaling pathways. Using a proteomic approach, Nishio *et al *identified marked differences in the phosphorylation status of specific nuclear proteins between drug sensitive and cis-diamminedichloro-platinum (II)-resistant cell lines [78]. Tyrosination-detyrosination is another PTM of tubulin, and Western blotting analysis has shown that tyrosinated tubulin is increased in paclitaxel-resistant breast cancer cells [79]. Histone proteins are subject to a range of PTMs in living cells. Deciphering of the histone code is hampered by the lack of analytical methods for monitoring the combinatorial complexity of reversible multisite modifications of histones, including acetylation and methylation. Mass spectrometry-based quantitative proteomic analysis of PTMs is a viable approach for functional analysis of candidate drugs, such as histone deacetylase inhibitors [80].

Since most novel therapeutic targets are proteins, proteomic analysis potentially has a central role in patient care. Understanding the molecular basis of tumor characteristics will usher a new era of individualized cancer therapy. Oncoproteomic analysis therefore represents a more direct way of investigating malignancy at the individual cancer patient level. Personalized management of cancer means the prescription of specific therapeutics that best suit for an individual patient and the type of tumor. Oncoproteomics will play an important role in the development of personalized cancer therapy. Molecular diagnostics influences cancer management in several ways that aid personalization. Oncoproteomics for cancer staging and personalization of therapy at the time of diagnosis could improve patient care. Application of pharmacogenetics will reduce the adverse effects of anticancer drugs. Cell/gene therapies, cancer vaccines, and RNA interference will facilitate the development of personalized cancer therapy [81,82].

The discovery of new highly sensitive and specific biomarkers for early disease detection and risk stratification coupled with the development of personalized therapies holds the key to future treatment of cancer. It is becoming clear that mapping the entire networks rather than individual markers may be necessary for robust diagnostics and tailoring of therapy. The emerging of oncoproteomics offers great promise for unraveling the complex molecular events of tumorigenesis, as well as those that control clinically important tumor behaviors such as metastases, invasion, and resistance to therapy. Functional imaging, biosensors, and sophisticated computational biology are having an unprecedented impact on the pharmaceutical industry [83]. Advanced proteomic platforms such as Orbitrap MS, Fourier transform ion cyclotron resonance MS, and protein microarrays can generate a rapid and high resolution portrait of the proteome  [84]. Emerging novel nanotechnology strategies to amplify and harvest tumor biomarkers *in vitro *or *in vivo *will greatly enhance our ability to discover and characterize molecules for early cancer detection, subclassification, and prognostic capability of current proteomic modalities [85]. New types of proteomic technologies combined with advanced bioinformatics are currently being used to identify molecular signatures of individual tumors based on protein pathways and signaling cascades. It is envisaged that analyzing the cellular circuitry of ongoing molecular networks will become a powerful clinical tool in cancer patient management [86].

Unlike information gathered by classical methods, high-throughput proteomic technologies can accurately inform clinicians on patient response to adjuvant therapy or those who will resist the effect of that therapy. Studies performed in cancer with high-throughput techniques have focused on tumor biology, prognosis, prediction of response to a few agents, and early diagnosis. Biomarker research has become a sign of the times, and the identified biomarkers may be used for clinical diagnostic or prognostic purposes. Biomarkers may also be used to help devising an optimal therapeutic treatment plan for different patient subsets and to monitor the effect of treatment. In this way, protein markers may be used to accelerate the speed and efficacy of clinical trials. Analysis of tumor-specific proteomic profiles permits better understanding of neoplasia development and the discovery of novel molecular targets for cancer therapy. Oncoproteomics plays an important role in cancer research and will have a significant impact on the development of future diagnostic and therapeutic products. In years to come, a serum or urine test for every phase of cancer may drive clinical decision making, supplementing or replacing currently existing invasive techniques.
